# Discovering Higher-Order Interactions Through Neural Information Decomposition

**DOI:** 10.3390/e23010079

**Published:** 2021-01-07

**Authors:** Kyle Reing, Greg Ver Steeg, Aram Galstyan

**Affiliations:** Information Sciences Institute, University of Southern California, Los Angeles, CA 90292, USA; gregv@isi.edu (G.V.S.); galstyan@isi.edu (A.G.)

**Keywords:** information theory, information decomposition, neural coding

## Abstract

If regularity in data takes the form of higher-order functions among groups of variables, models which are biased towards lower-order functions may easily mistake the data for noise. To distinguish whether this is the case, one must be able to quantify the contribution of different orders of dependence to the total information. Recent work in information theory attempts to do this through measures of multivariate mutual information (MMI) and information decomposition (ID). Despite substantial theoretical progress, practical issues related to tractability and learnability of higher-order functions are still largely unaddressed. In this work, we introduce a new approach to information decomposition—termed Neural Information Decomposition (NID)—which is both theoretically grounded, and can be efficiently estimated in practice using neural networks. We show on synthetic data that NID can learn to distinguish higher-order functions from noise, while many unsupervised probability models cannot. Additionally, we demonstrate the usefulness of this framework as a tool for exploring biological and artificial neural networks.

## 1. Introduction

At the center of statistics and machine learning is the idea that statistical regularities in data can be leveraged to build models and make predictions. Regardless of whether the model is specified by a simple parametric family, or learned incrementally using a deep neural network, the hope is to come away with a succinct description of the underlying relevant dependencies. Dependency in many settings commonly refers to the presence of pairwise interactions, which can be measured using metrics like the mutual information between two random variables. However, this is not the case in general, as any number of multivariate functions may exist among a group of random variables. Defining a proper measure of multivariate dependence is still an open and active area of research within information theory.

While standard measures of dependence may be sufficient for many problems, there are instances where we might expect higher-order interactions to play a critical role. For example, in microprocessors [1], many important computations are implemented with higher-order functions; such as XOR logic gates within full adders, and in error-correction schemes used for memory storage/retrieval. Testing whether this is also true in biological information processing (such as in neural circuitry [2] or in gene expression [3]) requires methods of probabilistic modeling which are sensitive to this type of dependence. It is expected that complex coordination at the level of individual units (such as neurons or genes) gives rise to collective behavior in the system, but the importance of each unit (or combinations of units) is often unknown.

Measures of multivariate correlation between units may produce a signature of the underlying computation [4], and understanding this signature may be a useful step towards understanding the full system. This has led many fields to explore ways in which the total information can be decomposed, so as to make the underlying causes more transparent. One result of this research direction was an expansion in the arsenal of multivariate mutual information (MMI) measures, including total correlation [5] and its generalization in the Cohesion measures of [6]. An alternative outcome of this pursuit is the Partial Information Decomposition [7], which is a promising framework for detailing the information that a group of source variables provide about a target. While theoretical explorations of this framework have yielded extensive results, practical application has only appeared in very limited settings [8], owing to the computational challenges associated with estimating the measures. The goal of this work is to begin investigating approaches to information decomposition which are usable in real-world (i.e., high-dimensional) settings.

The main contributions of the paper are as follows. (1) We introduce a new class of measures to determine the relevance of *k*th order interactions among a set of *n* variables (n>k). As a special case, these measures can be further decomposed to specify the information of order *k* that source variables *X* provide about a target variable *Y*. We arrive at the formalism for this decomposition by modifying existing measures of the total information. (2) We introduce an approach to estimate lower bounds on these measures based on an incremental strategy that builds upon representations of lower-order information. (3) We show that this approach leads to improved performance when the data contains higher-order functions. (4) We apply this machinery to simultaneous neural recordings from a salamander retina, and test the relevance of higher-order functions for understanding the neural code. (5) We take the first steps towards a theoretical comparison between our measures and PID on a suite of synthetic multivariate circuits. Section 3, Section 4 and Section 6 explore the theoretical properties and significance of our measures, while Section 4.1 and Section 5 focus on the practical implementation and challenges associated with probability estimation.

## 2. Related Work

Decomposition of higher-order interactions is an active area of research [7,9], with reviews of proposed measures appearing in [10,11]. While previous works focus on mathematically desirable properties of higher-order interaction measures, realistically these measures can be tractably evaluated only on small systems. Understanding synergies may be important for more complex systems, like the neural code [12], but existing methods do not scale to analyze even moderately-sized datasets. Our approach is inspired by the developing connection between latent factor modeling and rate-distortion theory [13,14]. Other recent work on tractable estimates of information measures focuses on mutual information [15,16], which may motivate improved bounds for NID. Recent work has also explored improving the interpretability of neural networks by reducing them to additive models with interactions of bounded degree [17]. While such approaches may be similar in spirit to our work, they assume that a network can learn the ground truth function, and that the order can be “read off” by evaluating its weights. The results of Table 1 suggest that this is unlikely for most networks if the data have truly higher-order functions. Although a full test of our measures’ efficacy in deconstructing artificial neural networks is outside the scope of this work, these results hint that our measures may be broadly applicable. In particular, an informative and tractable decomposition of a neural networks input/output mapping could unveil theoretical principles underlying learning and generalization, which are still severely lacking in the field. On a practical note, such advancements may also leave a positive impression on the field of fairness, which is leading the charge towards higher ethical standards in AI. Decomposing dependence runs counter to decades of black-box models, and may offer a path towards increased model transparency. Such transparency is desirable for both model understanding and risk management (as with a medical doctor attempting to interpret results that may save a patients life). Additionally, with a large amount of discriminatory bias present in our data and models, one may question the degree to which some biases remain elusive and hidden in plain sight due to their inherently higher-order nature.

## 3. MMI and Information Decomposition

In this work, we build upon two distinct notions of multivariate mutual information and its decomposition. The first is based on the decomposition of a particular measure of total information, known as the total correlation [5]. Under certain conditions, the total correlation can be generalized to preferentially weight information from higher-order subsets, as in the family of Cohesion measures [6] or the information-geometric measures of [18]. The second is the Partial Information Decomposition (PID) [7], which seeks to split up the mutual information *I*(*Y*:$X_{1:n}$) into a sum of multivariate contributions in $X_{1:n}$ about a target variable *Y*. Note that the former measures do not set aside a special variable *Y*, meaning the information they quantify is conceptually different from the PID. This dichotomy is ever-present in the information decomposition literature, as illustrated by work on entropy decompositions [19,20,21] that do not specify *Y*. For this reason, we refer to the PID as a directed measure of MMI (as it is only defined with respect to a target variable), and the former approaches as undirected measures. Most of the setup and experiments will focus on the undirected setting; however, the directed case is touched upon in Section 6.

### Total Correlation and Cohesion Measures

Consider a setting where a global measure of multivariate mutual information is defined among $X_{1:n}$ such that a single scalar ***c*** is returned. This is the case with the total correlation, which is frequently used as a measure of disentanglement in representation learning [22,23]. Total correlation is minimized when all variables are statistically independent and maximized when a bijective function exists between every pair of variables. One way to extend this measure is as follows,

(1) C(k)(X1:n):=1n−1k−1∑|A|=kH(XA)−H(X1:n)≥0

where the sum is taken over all nk subsets $X_{A}$ of size *k*. When k=1, this measure reduces to the total correlation: $TC\left(X\right)=\sum_{i=1}^{n}H\left(X_{i}\right)-H\left(X_{1:n}\right)$. This family of measures has been referred to as Cohesion measures [6], with each value of *k* providing more sensitivity to *k*th order dependence. For some *k*, $C^{\left(k\right)}$ is maximized when a bijective function exists between every pair of size *k* subsets, and no dependence exists in lower orders (≤k). These measures have also been studied as upper bounds on information-geometric measures of *k*th order information [18]. Ay et al. [18] also prove that these measures are monotone decreasing as a function of *k*, meaning the following difference is greater than or equal to zero,

(2) Ck−1||k:=C(k−1)−C(k)=1n−1k−2∑|B|=k−1H(XB)−1n−1k−1∑|A|=kH(XA)

Using this fact, we can write any Cohesion measure as a sum of local differences to obtain a more detailed decomposition of the total dependence:

(3) $$C^{\left(k\right)}=\sum_{l=k}^{n-2}C\left(l\;||\;l+1\right)+C^{\left(n-1\right)}$$

This allows us to report a list of up to n−1 non-negative values for the total correlation, rather than a single scalar which is less informative. This decomposition is more tractable than PID, as it does not require any additional optimization procedures and uses a fewer number of terms. However, this comes at the expense of a less expressive decomposition, which we will detail in forthcoming sections.

## 4. Neural Information Decomposition

For the local differences of Equation (2), the main computational challenges amount to estimating a combinatorial number of subset entropies. While modern solutions to unsupervised probability modeling [24,25,26] may provide approaches to estimate the entropy of these higher-order marginals, they do not provide practical solutions for dealing with such a large number of terms. One way forward is to subsample *l* subsets out of nk, and estimate the entropy on this reduced set. We include this approach as a baseline when l=n and show that it can work in certain cases. However, this strategy can severely underestimate the total information at order *k*, depending on the underlying distribution and how the subsets are selected.

Alternatively, one could propose a greedy strategy, wherein the estimation of higher-order subsets is based on the solution to lower-order subproblems. At a high level, we attempt to do this by constructing an incremental representation of the information at order k−1, and use this representation to inform modeling at order *k*. Our algorithm thus resembles a dynamic programming approach, or architectures used for sequential decision problems. Formally, we can rewrite Equation (2) in terms of a sum of conditional entropies, representing the growth from (k−1) to *k* order subsets (see Appendix A):

(4) C(k−1||k)=1kn−1k−2∑|B|=k−1H(XB)−1kn−1k−1∑i=1n∑|B|=k−1i∉BH(Xi|XB)

Instead of computing the first term outright, we can repeat the process of representing higher-order subsets using conditional entropy terms. If we consider many values of *k* (as in Equation (3)), we can move the first term of Equation (4) into C(k−2||k−1). Doing this recursively until k=1 leads to a simple formulation for the sum of local differences (proof in Appendix B):

(5) ∑l=1kC(l||l+1)=(k−1)k∑i=1nH(Xi)−1k∑l=2k1n−1l−1∑i=1n∑|C|=l−1i∉CH(Xi|XC)

Written this way, it becomes clear that these differences measure the incremental reduction in uncertainty for each variable $X_{i}$ as larger contexts of variables are considered. As it currently stands, this quantity is still computationally difficult, owing almost entirely to the combinatorial number of subsets. To alleviate some of this burden, we are interested in bounding the rightmost sum in a way that avoids computing so many terms. We propose to lower bound this sum using a representation $\phi_{-i}^{\left(l-1\right)}$ which is a function of all subsets $X_{C}$ of size l−1 that do not include *i*. To see why this is a lower bound, assume for the sake of illustration that $\phi_{-i}^{\left(l-1\right)}={\tilde{X}}_{C}$ such that $H\left(X_{i}|{\tilde{X}}_{C}\right)=\min H\left(X_{i}|X_{C}\right)$ (i.e., the representation copies the subset which maximally reduces uncertainty). Averaging over all subsets must lead to higher uncertainty than the minimum, implying the representation is a lower bound. Taking a Monte Carlo estimate of the expectation in each entropy term (where *M* is the total number of samples) yields an expression for the differences in terms of conditional log-likelihood.

(6) −1k∑l=2k1n−1l−1∑i=1n∑|C|=l−1i∉CH(Xi|XC)≥1kM∑s=1M∑l=2k∑i=1nlogp(Xi(s)|ϕ−i(l−1))

Note that if we start from k=1 and build up to larger values of *k*, each local difference only needs to compute *n* terms if the representations ϕ are known. We will discuss different ways of constructing ϕ in the next section. The goal is to maximize this term—or equivalently, minimize its negative value—in order to represent information up to order *k*. The final objective can thus be seen as minimizing a weighted sum of negative log-likelihoods for each $X_{i}$, conditioned on summarized contexts from different orders. Estimating the quantities in Equation (6) defines the core of our Neural Information Decomposition (NID) approach, with Neural in this context referring to the use of powerful neural networks. While this is the first work to combine the pragmatism of neural density estimation with ideas from information decomposition, we expect many future works to explore and refine this concept. NID can thus be seen as a descriptive term, referring to space of solutions attempting to bridge the gap between these fields.

### 4.1. Choosing the Representations ϕ

**Figure 1:**
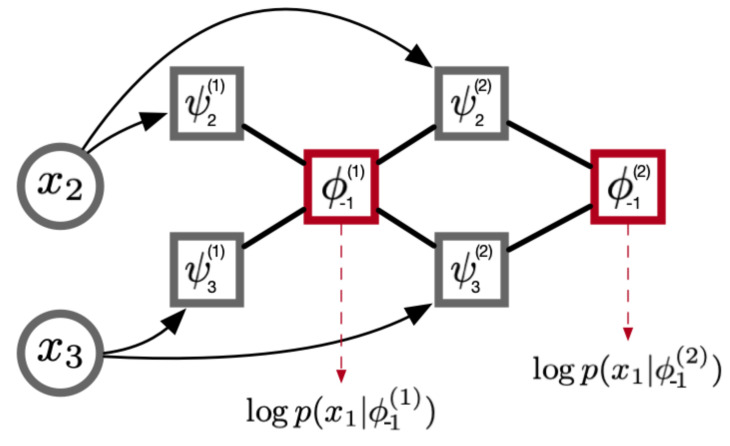
Above is a visual representation of the steps for our algorithm. In the three variable case, we predict each variable using contexts of size 1 and 2. These contexts are generated incrementally by first generating independent features (ψ, learned by a neural network), then combining/selecting (ϕ, a constrained aggregation such as mean) those that are most informative. This produces incremental likelihood estimates for $x_{1}$, and the same procedure is done for each $x_{i}$ separately.

While our experiments only focus on parameterizing the log-probabilities of Equation (6) under a Bernoulli or Categorical distribution, a Gaussian log-likelihood or normalizing flow [27,28] could be used in the continuous case. The architecture underlying NID is made up of two simple components: we first take an existing lower-order representation $\phi_{-i}^{\left(k-1\right)}$ (or learnable bias at the start for $\phi_{-i}^{\left(0\right)}$) and compute statistics $\psi_{j\neq i}^{\left(k\right)}=f\left(\phi_{-i}^{\left(k-1\right)},X_{j\neq i}\right)$ for each $X_{j}\neq X_{i}$ using a 2-layer feedforward network. This process generates new candidate representations by expanding the context with information from one additional variable ($X_{j}$). We then use these statistics to build new higher-order representations $\phi_{-i}^{\left(k\right)}=g\left(\psi_{j\neq i}^{\left(k\right)}\;\forall j\neq i\right)$ through a summary or pooling operation over all candidate statistics. This entire process is represented visually in Figure 1 below on a simple three variable example. The choice of summary function is difficult, as preserving exclusively *k*th order information in $\phi_{-i}^{\left(k\right)}$ requires appropriate constraints on $g(\psi_{j\neq i}^{\left(k\right)}\;\forall j\neq i)$. While these constraints can be achieved with certain operations—such as min/max pooling—we found them too restrictive to learn meaningful representations in practice. For most experiments, we chose to use mean pooling as a representation function. While this pooling can learn to represent information between statistics which is above the desired value of *k*, the information is heavily constrained due to the linearity of the function. Although the one-to-one correspondence between layer number and order of information is lost under this operation, we provide evidence that it does not drastically deviate from the ground truth order. In the following section, we will compare this architecture to baselines from sequence modeling (LSTMs, RNNs, and GRUs), generative models (variational autoencoders), and autoregressive density estimators.

## 5. Experiments

To facilitate reproducibility, the code and scripts for running experiments can be found in the supplementary. Additionally, we have included information about model architectures and metrics in Appendix F. Our experiments aim to test whether the NID architecture and the objective of Equation (6) can be used to effectively model higher-order interactions. In comparison to standard approaches, NID provides a more detailed description of where the information exists in a distribution, which may be desirable when attempting to understand certain systems. We show that NID performs as well or better on synthetic data with higher-order interactions, while simultaneously producing an accurate and expressive decomposition.

### 5.1. Distinguishing Error-Correcting Codes From Noise

In our first set of experiments, we introduce two synthetic datasets that only contain higher-order dependencies (i.e., all lower order information is indistinguishable from noise). The first of these is based on the high-dimensional parity distribution. We generate data by drawing uniform samples from the $2^{n-1}$ hypercube, then add an additional random variable corresponding to the parity of the previous n−1. In each dataset, we generated roughly 100,000 samples, and used a 70/30 train/validation split. Even this simple example is deceptively challenging from a learning perspective, with theoretical results for related problems, such as learning parity with noise [29], the classical XOR problem in supervised learning [30], overparameterization in XOR detection [31], and the depth of computation required for learning higher-order functions [32]. Recent work has also illustrated the practical failure of standard deep networks to efficiently learn/represent the parity function [33].

In Table 1, we report values for the negative log-likelihood on four different sizes of parity data, with mean and deviation across 5 random initializations. Each of the approaches in the top half of the table attempt to model the joint density of the data, with the first three being sequence models (RNN, LSTM, and GRU) and the remaining being autoregressive density estimators (MADE, MADE-U, and MADE-S). As n−1 of the variables are indistinguishable from noise, we only report the negative log-likelihood for the last variable in the sequence/autoregressive prediction. This value represents how well a given architecture can reproduce the parity mapping for a fixed ordering of the variables. If the model learns the correct functional relationship, it should report 0 nats (appearing as bolded values in the table), and if cannot distinguish the relationship from noise, it should report 0.693 nats. For the sequence models, we attempt to predict the next variable in the sequence from the current variable and some cumulative latent representation. The autoregressive models are all MADE networks [25] which parameterize the logits of Bernoulli random variables. We explored two ways of incorporating multiple autoregressive orderings into MADE, by either (a) ensembling the outputs of unique MADE networks for each ordering (MADE-U), or (b) using a single MADE network shared across all orderings (MADE-S). In both cases, we selected a linear number of orderings at random, since parity is symmetric in the ordering of variables. Note that doing this implies MADE-U and MADE-S test the naive baseline introduced in Section 4 when k=n. For values of k<n there is no structure to learn, and each model correctly returns the log-likelihood of random noise. We found that the performance of most models degraded in the large parity regime. For 15 and 20 parity, none of the sequence models were able to identify the ground truth relationship. Note that we avoid injecting additional supervision in these models, such as providing variable length sequences, introducing symmetry priors, or predicting the cumulative parity. The autoregressive models did much better in these cases, with most of the variants converging to the ground truth. Adding multiple orderings (MADE-U and MADE-S) helped the autoregressive models in the higher-dimensional cases. While both extensions performed similarly on all experiments, MADE-U used a separate model for each of the *n* orderings, making it extremely inefficient in the number of parameters.

In the bottom half of Table 1, we compare models that lower bound the joint density. The first two models are generative models (Autoencoders and Gaussian Variational Autoencoders), and the remaining are variants of the NID algorithm. While both of these approaches optimize a lower bound, they differ in how they measure the quality of a learned representation. To discourage memorization, autoencoders create a bottleneck in the architecture and often add noise to the compressed intermediate representation. However, this is not a hard constraint on the flow of information through the network, and autoencoders are still prone to memorization. In contrast, NID attempts to predict $X_{i}$ using only information from other variables $X_{j}$, which prevents the network from passing information about $X_{i}$ directly to the output. Unlike the sequence and density models, which could only meaningfully predict a single variable, both of these approaches can potentially predict every variable. For this reason, we report the sum of negative log-likelihoods for all variables. By Equation (6), we see that NID contains a different term for reconstruction at each order *k*. We chose k=5 for these experiments (i.e., the NID network has 5 layers of incremental likelihood estimation), and show the reconstruction at the final value of *k* for the sake of comparison. In the case of parity, we found a larger number of layers to be detrimental to performance for two reasons: (1) early layers have nothing to predict (by design of the experiment), leading to an inefficient utilization of model parameters and computation; (2) early representations heavily influence future computations, and networks with a large number of layers were prone to unstable learning dynamics. This was especially noticeable in parity, as early layers do not have a salient gradient signal to follow during training. The left half of Table 2 below shows how the differences between intermediate layers compare to the ground truth decomposition for 5-parity. Recall that the local differences of Section 4 measure the incremental change in likelihood when increasing the subset size, and Equation (6) measures the change in likelihood when utilizing contextual representations of increasing size. Using mean as our representation function allowed the network to detect structure at earlier contextual size (between layers 3 and 4) than the ground truth subset size of 4 and 5.

We found that NID consistently outperforms the autoencoders in terms of reconstruction. To further verify that the network is learning to identify the correct relationships instead of memorizing the input, we freeze the weights/gradients of a trained NID model and feed its outputs into a small feed-forward MLP (NID + MLP). By the nature of parity, if the ground truth function is known for at least one variable, then a simple linear function can extend this prediction to all other variables. We see that this is indeed the case for NID, as NID + MLP is able to achieve near perfect reconstruction. If we attempt to do the same thing with the output of the autoencoders, we do not observe any change in the overall reconstruction. This implies that the autoencoders are memorizing noise information to improve reconstruction, rather than learning useful or meaningful features from the data.

The second dataset is based on error-correcting codes (MDS codes [34]), which can be thought of as a generalization of the parity function. In MDS codes, the dependence exists among any subset of greater than *k* variables, while all subsets of size *k* are independent. Each conditional marginal in an MDS code is parameterized by a Categorical distribution with discrete support q=n, compared to q=2 for parity. As with the previous synthetic dataset, we compare the aforementioned approaches on this data, with results appearing in Table 3. As a slight change of notation, the bolded values in this Table represent the best approach (in terms of NLL) for each column/dataset. These data are strictly harder than the parity problem, and many of the approaches did poorly in comparison. In particular, both of the autoencoders were unable to distinguish structure from noise in any of the datasets. Although NID was able to perform well in some cases, we observed a large amount of variability and sensitivity to initial conditions on this data in comparison to parity (as seen in the deviation for NID across almost every MDS experiment). However, when freezing and combining with an MLP (as with the parity experiments), the variability reduces substantially. This can be explained as follows. The reported values correspond to a sum of independent predictors for each $X_{i}$ based on contexts of increasing order. If even a single one of these predictors learns the true function, then the MLP can use it to predict all other variables. High variability in the standard setting implies that the true function is not always learned for each $X_{i}$, whereas low variability in NID + MLP implies the true function is learned consistently at least once.

### 5.2. Higher-Order Interactions in the Neural Code

In their work on using maximum entropy methods to test for collective behavior in the neural code, the authors of [35] used data collected from simultaneous recordings of a salamander retina. These data were recently made open source [36], and they contain a description of the setting under which experiments were conducted, along with the pipeline for data collection. To summarize some of the key points, the data consists of neural spike trains recorded from salamander retinal ganglion cells, with potentials binned at 20 ms (meaning any activation of a single neuron within the time window is recorded as 1). In total, there are 160 neurons and approximately 300,000 samples gathered over a period of 2 h. This data is one of very few public datasets that contain simultaneous measurements of neurons from a multi-electrode array (MLA). Simultaneous recording is critical for studying the neural code, as neuron co-activation (population coding) and temporal locality (rate coding) are the main contenders for how information is encoded in the brain.

**Figure 2:**
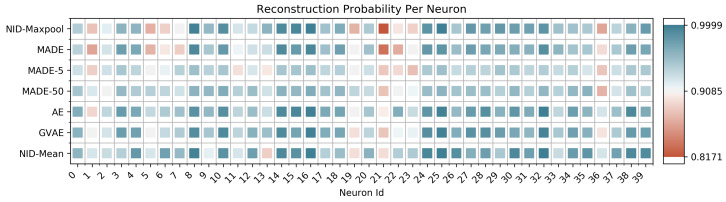
Reconstruction error for individual neurons across multiple approaches. MADE corresponds to a standard autoregressive model without any reorderings. MADE-5 and MADE-50 are variants of MADE-S with k=5 and k=50, respectively. AE and GVAE correspond to Autoencoder and Gaussian Variational Autoencoder. The neurons are colored such that dark blue is the highest (0.999) and dark red is the lowest (0.817) likelihood reported on a held out test set.

To further explore the collective hypothesis, we applied models from the previous section to this data in a number of different ways. The left side of Table 4 shows the negative log-likelihood obtained by autoencoders and Gaussian variational autoencoders. In addition to the standard MLP architecture used in previous sections, we test how well these models do when using a Convolutional architecture. On the right side of Table 4, we use MADE-S and parameterize a linear number of subsets using a sliding window of size *k* (the baseline mentioned at the beginning of Section 4). Note that, unlike the parity experiment, it makes sense in this context to use k<n. The numbers reported in Table 4 were from the *k* which produced the best likelihood, which happened to be 50 in our experiments (shown in Figure 2 as MADE-50). We compare the above approaches against NID using mean pooling (NID-Mean) and max pooling (NID-Maxpool) as the representation function. Additionally, in Table 5 we report the intermediate reconstruction of NID-Mean at each layer (as in Table 2). If we plot the negative log-likelihood for each neuron across different methods—or when blocks of size *k* are used in MADE-S, an average over the *k* appearances of this neuron in the loss—we observe that many neurons are almost perfectly predictable, with a select few responsible for the gap in reconstruction. Figure 2 shows a random selection of 40 neurons, with plots for the remaining 120 appearing in Appendix E.

Even for a relatively small value of k=5 (shown in Figure 2 as MADE-5), NID performs comparably or better than both autoregressive models and autoencoders, and often does better at modeling critical neurons. This result lends itself to the following interpretation. While lower-order dependency might be global among the collection of neurons (for example, through redundant activations), higher-order dependency is likely not a global property. This observation is supported by the capability of all approaches to almost perfectly model most neurons. At the local level, some neurons are difficult to completely predict with standard approaches, but can be predicted with NID-Mean. This might imply that the activity of certain neurons depends on higher-order functions among neighboring neurons (or, more generally, functions for which our approach is more capable of modeling). Given what is known about the Neurophysiology of the retina, these results may not be so surprising. Most of the neuronal processing in early visual areas is thought to be feedforward, with a sparse collection of inhibitory cell types (such as amacrine and horizontal cells) that play a role in defining spatial receptive fields [37]. The neurons that are more difficult to predict may correspond to these laterally interconnected neurons, although there is no way for us to test this hypothesis on the current data. One interesting path forward for future work would be analyze data in which cell types are known, to see if they could be differentiated purely by their activation patterns (as opposed to their connectivity structure [38], or the use of imaging). Additionally, evaluation of different brain regions (other than early sensory processing) may yield more interesting (higher-order) information profiles.

## 6. Directed Multivariate Information of Order-k

In Neuroscience, it is often desirable to quantify the effect of a stimulus on some response, such as the firing of a particular group of neurons [39,40]. The partial information decomposition (PID) of [7] was proposed with this task in mind, and has since sparked an active research community focused on how multivariate information about a target can be decomposed into a sum of non-negative quantities. In the case of three variables (two source variables and one target), the PID yields four individually meaningful terms:

(7) $$I\left(Y:X_{1,2}\right)=R\left(Y;X_{1,2}\right)+S\left(Y;X_{1,2}\right)+U\left(Y;X_{1}\right)+U\left(Y;X_{2}\right).$$

for appropriate definitions of redundant R(·) [41], synergistic S(·) [10], and unique U(·) [42] information. Much of the recent work in this community is aiming to find a consistent definition of these terms [43,44], solving theoretical issues regarding the incompatibility of local positivity and the so-called identity axiom [45].

One issue that limits the applicability of this decomposition is computational tractability. When moving beyond the three variable case, the number of terms grows super exponentially in the number of variables based on the (n−1)th Dedekind number (i.e., the total number of monotone Boolean functions among *X*) [7]. Additionally, each term often requires optimizing over some convex polytope in the space of probability distributions [46,47], which is non-trivial in all but the smallest cases. Exploring a middle-ground between tractability and expressibility is necessary if methods of information decomposition are to be used on high-dimensional data.

### 6.1. Directed Local Differences

Although Equations (2) and (3) make a positive step towards a more tractable decomposition, they are not immediately comparable to the *directed* formulation of standard information decomposition approaches. Below, we introduce a novel extension of these measures by showing how a measure of *directed* MMI about *Y* of order *k* can be obtained from the *undirected* setting. We can express Cohesion for a particular value of *k* over the joint distribution containing *X* and *Y* as

(8) C(k)(X1:n,Y)=1nk−1∑|B|=k−1H(Y,XB)+1nk−1∑|A|=kH(XA)−H(Y,X1:n)

Here, we assume that the total number of variables (including *Y*) is n+1. This expression can be rewritten as a Cohesion measure that only depends on *X*, a weighted local difference from k−1 to *k*, and a remainder of terms which we label $C_{Y}^{\left(k\right)}$ (proof in Appendix D).

(9) C(k)(X1:n,Y)=C(k)(X1:n)+CY(k)(X1:n)+(k−1)nC(k−1||k)CY(k)(X1:n):=1nk−1∑|B|=k−1H(Y|XB)−H(Y|X1:n).

By representing $C^{\left(k\right)}\left(X_{1:n},Y\right)$ and $C^{\left(k\right)}\left(X_{1:n}\right)$ in terms of their local differences, we can thus define a local difference of consecutive *directed* MMI in a similar way to Equation (2).

(10) CY(k−1||k):=CY(k−1)−CY(k)=1nk−1∑|B|=k−1H(Y|XB)−1nk∑|A|=kH(Y|XA)

Each local difference is considered as a measure of information at order *k* in the source variables *X* about a target variable *Y*. These measures are non-negative by the data processing inequality; namely, going from subsets of size k−1 to *k* cannot make *Y* less predictable on average. Additionally, summing over all values of *k* from 0 to *n* (as in Equation (3)) yields a decomposition of the mutual information:

(11) $$I\left(Y:X\right)=\sum_{k=1}^{n}C_{Y}\left(k-1||k\right)=H\left(Y\right)-\frac{1}{n}\sum_{i}H\left(Y|X_{i}\right)+\frac{1}{n}\sum_{i=1}^{n}H\left(Y|X_{i}\right)\cdots -H\left(Y|X_{1:n}\right)$$

We now compare these measures to a number of existing approaches on a set of canonical synthetic examples.

### 6.2. Canonical Comparisons

One of the main benefits of these measures over existing approaches is that they can be expressed entirely in terms of subset entropies. This means that no additional optimization is needed if the ground truth probabilities are known. However, if the measures are not in sufficient agreement with various axiomatic properties, their value may not accurately reflect the ground truth interactions. To check if this is the case, we compare the directed measures in the three variable (bivariate) case on a variety of small synthetic tests.

These tests appear frequently in the information decomposition literature when probing the axiomatic properties of a measure. Table 6 shows the results of this comparison, where we interpret $C_{Y}\left(0||1\right)$ as a measure of redundancy and $C_{Y}\left(1||2\right)$ as a measure of synergy. While the number of potential measures to compare against is large, we stick to two classical measures with close ties to the PID: the first being the shared ($\tilde{SI}$, redundant) and complementary ($\tilde{CI}$, synergistic) information associated with the BROJA measure [42], and the second being the original $I_{min}$ measure of redundancy from [7]. Surprisingly, the values of our measures are consistent with classical lattice-based measures on a number of examples. However, certain examples (ex: Unique) reveal limitations that come about due to the reduced number of terms present in our decomposition.

Despite its use as a measure of redundancy in the previous examples, $C_{Y}\left(0||1\right)$ actually captures all first-order information about the target *Y*. This implies that first-order redundancy and unique information are confounded into a single scalar. To illustrate this in more detail, we show in Appendix C how certain axioms for redundancy measures are violated by our measures. The fact that unique information becomes confounded with other relevant quantities at a particular order *k* is an immediate and inescapable consequence of seeking out a tractable decomposition with fewer terms.

To further explore connections to PID, we compare results obtained from our measures in the tripartite case to the maxent/optimization approach of Makkeh et al. [48]. The circuits we tested on include three variable extensions of the Redundant, XOR, and AND gates from Table 6, as well as the sum gate ($Y=X_{1}+X_{2}+X_{3}$) and the copy gate ($Y=(\left(X_{1},X_{2}\right),\left(X_{1},X_{3}\right),\left(X_{2},X_{3}\right))$) described in [49]. To start, there are a number of striking similarities between values across approaches, despite fundamental differences in the measures. The measure of redundancy (R) in MAXENT-3-PID is consistent with the first local difference $C_{Y}\left(0||1\right)$ on all examples except the Copy gate (see Table 7). However, the redundancy value that MAXENT-3-PID obtains on this gate (0) is not consistent with values obtained by existing measures [49], while the NID estimates are. Another curious observation is that the value for synergy in MAXENT-3-PID is often equal to the sum of the last two differences $C_{Y}\left(1||2\right)+C_{Y}\left(2||3\right)$ (as in the case of the Sum and And gates). We are unsure of the significance (if any) behind this fact, but it may prove to be an interesting starting place for further investigation.

## 7. Conclusions

In this work, we proposed Neural Information Decomposition, a framework for representing and decomposing the contribution of different orders of functional dependence to the total information. Motivated by the theoretical setup of Equation (5), we introduced an architecture to incrementally build representations of information at a particular order *k*. We demonstrated the applicability of this framework on synthetic examples constructed to contain higher-order functions and on neural spike train data to test for signs of collective computation in the neural code. We believe this work represents a positive first step towards a tractable information decomposition, but there is still room to improve (especially regarding the architecture and choices of representation function in Section 4.1). In future work, we hope to expand the scope of applications to include the directed setting of Equation (10). In particular, we are interested in measuring the *k*th order information in neurons which is informative about some stimulus. This evaluation can also be applied to artificial neural networks to try to make sense of their internal computations. Additionally, we plan on further exploring the implications of results we have obtained on biological neural networks, and what it can tell us about how the brain processes information.

## Figures and Tables

**Table 1:** Negative log-likelihood for various models on parity data.

Model	5-Parity	10-Parity	15-Parity	20-Parity
RNN	0.01±0.01	0.04±0.01	0.693±0.03	0.693±0.02
LSTM	0.05±0.05	0.02±0.01	0.693±0.01	0.693±0.01
GRU	0.01±0.01	0.01±0.01	0.693±0.01	0.693±0.01
MADE	0.02±0.01	0.03±0.02	0.05±0.2	0.475±0.15
MADE-U	0.01±0.01	0.02±0.01	0.04±0.01	0.08±0.03
MADE-S	0.04±0.01	0.03±0.01	0.03±0.01	0.09±0.04
Autoencoder	2.79±0.67	6.5±0.43	8.18±1.2	12.57±1.2
Gaussian VAE	3.24±0.14	6.15±0.22	10.38±0.01	13.85±0.01
NID	0.01±0.01	2.28±0.6	4.78±1.1	11.12±1.4
NID + MLP	0.01±0.01	0.01±0.01	0.03±0.01	0.05±0.02

**Table 2:** Local differences for 5-Parity (Left) and MDS (*n* = 7, *k* = 3) (right) in nats.

Order (k)	C(1||2)	C(2||3)	C(3||4)	C(4||5)	C(1||2)	C(2||3)	C(3||4)	C(4||5)
Ground Truth	0	0	0	0.693	0	0	3.4	2.04
NID	0	0	0.648	0.409	0	1.49	0.77	0.01

**Table 3:** Negative log-likelihood for various models on MDS data.

Model	MDS (*n* = 7, *k* = 3)	MDS (*n* = 7, *k* = 5)	MDS (*n* = 11, *k* = 3)	MDS (*n* = 11, *k* = 5)
RNN	5.72±0.04	13.62±0.01	7.30±0.02	26.36±0.01
LSTM	5.92±0.02	4.56±0.07	7.40±0.06	26.36±0.01
GRU	5.86±0.07	13.63±0.01	26.36±0.01	26.36±0.01
MADE	4.27±0.11	4.39±0.16	10.53±0.17	12.47±0.32
MADE-U	4.61±0.8	4.63±0.4	10.85±0.24	12.25±0.27
MADE-S	4.52±0.13	4.22±0.12	11.36±0.22	11.97±0.32
Autoencoder	13.63±0.01	13.64±0.01	26.38±0.01	26.36±0.02
Gaussian VAE	13.62±0.01	13.62±0.01	26.37±0.01	26.37±0.01
NID	1.67±0.42	6.47±2.12	6.48±2.52	14.46±4.14
NID + MLP	0.04±0.02	0.29±0.07	0.01±0.01	0.35±0.05

**Table 4:** Negative log-likelihood for various methods on salamander retinal ganglion data.

AE-MLP	AE-Conv	VAE-MLP	VAE-Conv	MADE-S	NID-Maxpool	NID-Mean
15.85	12.64	17.78	19.16	18.10	18.89	11.61

**Table 5:** Local differences between NID layers on salamander retinal ganglion data.

Order (k)	C(1||2)	C(2||3)	C(3||4)	C(4||5)	C(5||6)
NID	15.23	53.42	18.2	10.44	2.08

**Table 6:** For explanation of the generating process for each test case, see in [42].

Tests	$C_{Y}\;\left(0||1\right)$	$C_{Y}\;\left(1||2\right)$	$\tilde{\mathrm{SI}}$	$\tilde{\mathrm{CI}}$	$I_{\min}$
Redundant	1	0	1	0	1
Unique	1	1	0	1	1
XOR	0	1	0	1	0
AND	0.311	0.5	0.311	0.5	0.311
Red-XOR	1.5	1.5	1	1	1
XOR-AND	0.5	1	0.5	1	0.5

**Table 7:** Comparison between directed local differences and terms in the tripartite PID, as computed by the MAXENT-3-PID software of Makkeh et al. [48].

Tests	$C_{Y}$ (0||1)	$C_{Y}$ (1||2)	$C_{Y}$ (2||3)	R	U ($X_{1}$)	U ($X_{2}$)	U ($X_{3}$)	U ($X_{1,2}$)	U ($X_{1,3}$)	U ($X_{2,3}$)	S
Red	1	0	0	1	0	0	0	0	0	0	0
XOR	0	0	1	0	0	0	0	0	0	0	1
3-AND	0.1379	0.1556	0.25	0.1379	0	0	0	0	0	0	0.4056
SUM	0.311	0.5	1	0.311	0	0	0	0	0	0	1.5
Copy	1	1	1	0	1	1	1	0	0	0	0

## Data Availability

Data sharing not applicable.

## Appendix A.

**Proof** **of** **Equation (4)**. This proof draws from the proof of Proposition 3.2 in [18]. Namely, we can replace the second term of Equation (2) with

1n−1k−1∑|A|=kH(XA)=1kn−1k−1∑i=1n∑|B|=k−1i∉BH(Xi|XB)+n−(k−1)kn−1k−1∑|B|=k−1H(XB)

Substitution leads to the expression of Equation (4). □

## Appendix B.

**Proof** **of** **Equation (5)**. Leveraging results from Equation (4):

C(k−2||k−1)+C(k−1||k)=1n−1k−3∑|C|=k−2H(XC)−1n−1k−2∑|B|=k−1H(XB)+1kn−1k−2∑|B|=k−1H(XB)−1kn−1k−1∑i=1n∑|B|=k−1i∉BH(Xi|XB)=1n−1k−3∑|C|=k−2H(XC)−(k−1)kn−1k−2∑|B|=k−1H(XB)−1kn−1k−1∑i=1n∑|B|=k−1i∉BH(Xi|XB)=2kn−1k−3∑|C|=k−2H(XC)−1kn−1k−2∑i=1n∑|C|=k−2i∉CH(Xi|XC)−1kn−1k−1∑i=1n∑|B|=k−1i∉BH(Xi|XB)

Repeating this procedure by continuing to add local differences all the way down to the first values of *k* leads to the expression in Equation (5). □

## Appendix C. Confounding Redundancy and Unique Information

We can observe the consequences of treating $C_{Y}\left(0||1\right)$ as a measure of redundancy when unique information is present. Kolchinsky [49] discusses a number desirable axioms which a measure of redundancy should satisfy, including *Symmetry*, *Self Redundancy*, the *Deterministic Equality*, and *Monotonicity*. We will briefly show that $C_{Y}\left(0||1\right)$ satisfies these axioms when no Unique information is present, and fails otherwise.

**Symmetry:** The symmetry axiom states that redundancy does not change under permutation of the input variables $X_{1:n}$, which is trivially true for $H\left(Y\right)-\frac{1}{n}\sum_{i=1}^{n}H\left(Y|X_{i}\right)$.

**Self Redundancy:** The self redundancy axiom states that redundancy is equal to the mutual information in the case of one input variable, which is also trivially true: $H\left(Y\right)-\frac{1}{1}H\left(Y|X_{1}\right)=I\left(Y:X_{1}\right)$.

**Deterministic Equality:** The deterministic equality axiom states that adding a source variable will not change the redundancy if the new source is a deterministic function of an existing source. In the simplest case, we can see that $H\left(Y\right)-H\left(Y|X_{1}\right)-H\left(Y\right)+\frac{1}{2}H\left(Y|X_{1}\right)+\frac{1}{2}H\left(Y|f\left(X_{1}\right)\right)=0$. More generally, if we take the difference between $C_{Y}\left(0||1\right)$ for the *n* and n+1 variable case (assuming $Z=f(X_{i})$ is the added source), we get

$$\begin{array}{cc} \; & H\left(Y\right)-\frac{1}{n}\sum_{i=1}^{n}H\left(Y|X_{i}\right))-H\left(Y\right)+\frac{1}{n+1}\sum_{i=1}^{n}H\left(Y|X_{i}\right)+\frac{1}{n+1}H\left(Y|Z\right)\\ \; & =\frac{1}{n+1}H\left(Y|Z\right)-\frac{1}{n(n+1)}\sum_{i=1}^{n}H\left(Y|X_{i}\right) \end{array}$$

If *Z* is as informative about *Y* as the mean over all existing sources (ex: when no unique information exists and all source variables are deterministic functions of each other), then the above quantity evaluates to 0 and the axiom is satisfied. If *Z* is more informative about *Y* (alt. less), the quantity evaluates to a negative number (alt. positive), meaning the axiom is not satisfied in most cases.

**Monotonicity:** The monotonicity axiom states that the redundancy decreases or stays the same as new source variables are added. Interestingly, a similar axiom holds in the other direction (i.e., increases or stays the same) for measures of union information [49]. Using the results detailed in the Deterministic Equality, we know that the monotonicity axiom is satisfied when all sources are redundant, or when *Z* is less informative about *Y* than the average over existing sources.

## Appendix D.

**Proof** **of** **Equation(9)**.

C(k)(X1:n,Y)=1nk−1∑|B|=k−1H(XB)+1nk−1∑|B|=k−1H(Y|XB)+1nk−1∑|A|=kH(XA)−H(Y|X1:n)−H(X1:n)=1n−1k−1∑|A|=kH(XA)−H(X1:n)−(k−1)nn−1k−1∑|A|=kH(XA)+(k−1)nn−1k−1∑|B|=k−1H(XB)+1nk−1∑|B|=k−1H(Y|XB)−H(Y|X1:n)=C(k)(X1:n)+(k−1)nC(k−1||k)+CY(k)(X1:n)

□

## Appendix E. Additional Neuron Plots

These are the plots for the remaining 120 out of 160 neurons in the salamander retina dataset:

## Appendix F. Model Architectures

**Sequence Models:** All sequence models were implemented in Tensorflow using standard keras models. For the parity data, all sequence models used the following configurations: (a) 5 parity: 10 neurons, 1 layer; (b) 10 parity: 20 neurons, 1 layer; (c) 15 parity: 30 neurons, 2 layers; (d) 20 parity: 50 neurons, 3 layers.

For the MDS data, all sequence models used the following configurations: (a) 7/3 MDS: 30 neurons, 1 layer, 20-dimensional embedding; (b) 7/5 MDS: 50 neurons, 2 layer, 20-dimensional embedding; (c) 11/3 MDS: 30 neurons, 2 layers, 20-dimensional embedding; (d) 11/5 MDS: 50 neurons, 3 layers, 20-dimensional embedding.

**Autoregressive Models:** All autoregressive models were implemented in Tensorflow using the tensorflow probability library and default MADE network. For the parity data, all autoregressive models used the following configurations: (a) 5 parity: 10 neurons, 1 layer; (b) 10 parity: 20 neurons, 1 layer; (c) 15 parity: 30 neurons, 2 layers; (d) 20 parity: 50 neurons, 3 layers.

For the MDS data, all autoregressive models used the following configurations for the MADE network: (a) 7/3 MDS: 35 neurons, 2 layer; (b) 7/5 MDS: 50 neurons, 3 layer; (c) 11/3 MDS: 55 neurons, 2 layers; (d) 11/5 MDS: 55 neurons, 3 layers

**MLPs:** For autoencoders based on MLPs, we used a 6-layer architecture for both encoder and decoder. Activations between each layer were ReLU, with the smallest layer (output of the encoder) equaling 20 neurons for the neural data, and smaller than the input size for synthetic data (2 for 5-parity, 3 for 10-parity, and 5 for 20-parity). For the MLP used after autoregressive models, we used a simple 2-layer MLP with ReLU activations.

**Convolutional:** All autoencoders using a convolutional architecture on the salamander retina dataset used the following architecture.

32 filt, 2 × 2 kernels, stride 1, pad 0, batchnorm, ReLU
16 filt, 3 × 3 kernels, stride 2, pad 1, batchnorm, ReLU
8 filt, 2 × 2 kernels, stride 2, pad1, batchnorm, ReLU
4 filt, 3 × 3 kernels, stride 2, pad 1, batchnorm, ReLU
1 filt, 1 × 1 kernels, stride 1, pad 1, linear activation
